# Bacterial co-infection of the respiratory tract in ventilated children with bronchiolitis; a retrospective cohort study

**DOI:** 10.1186/s12879-019-4468-3

**Published:** 2019-11-06

**Authors:** Hanke M. G. Wiegers, Lisa van Nijen, Job B. M. van Woensel, Reinout A. Bem, Menno D. de Jong, Job C. J. Calis

**Affiliations:** 1 Pediatric Intensive Care Unit, Emma Children’s Hospital, Academic Medical Center, Meibergdreef 9, 1105 AZ Amsterdam, The Netherlands; 2 Department of Medical Microbiology, Academic Medical Center, Amsterdam, The Netherlands

**Keywords:** Coinfection, Bacterial infections, viral bronchiolitis, Artificial respiration, Child, Ventilator associated pneumonia

## Abstract

**Background:**

Viral bronchiolitis is the most common cause of respiratory failure requiring invasive ventilation in young children. Bacterial co-infections may complicate and prolong paediatric intensive care unit (PICU) stay. Data on prevalence, type of pathogens and its association with disease severity are limited though. These data are especially important as bacterial co-infections may be treated using antibiotics and could reduce disease severity and duration of PICU stay. We investigated prevalence of bacterial co-infection and its association with disease severity and PICU stay.

**Methods:**

Retrospective cohort study of the prevalence and type of bacterial co-infections in ventilated children performed in a 14-bed tertiary care PICU in The Netherlands. Children less than 2 years of age admitted between December 2006 and November 2014 with a diagnosis of bronchiolitis and requiring invasive mechanical ventilation were included. Tracheal aspirates (TA) and broncho-alveolar lavages (BAL) were cultured and scored based on the quantity of bacteria colony forming units (CFU) as: co-infection (TA > 10^^5^/BAL > 10^^4^ CFU), low bacterial growth (TA < 10^^5^/BAL < 10^^4^ CFU), or negative (no growth). Duration of mechanical ventilation and PICU stay were collected using medical records and compared against the presence of co-infection using univariate and multivariate analysis.

**Results:**

Of 167 included children 63 (37.7%) had a bacterial co-infection and 67 (40.1%) low bacterial growth. Co-infections occurred within 48 h from intubation in 52 out 63 (82.5%) co-infections. *H.influenza* (40.0%), *S.pneumoniae* (27.1%), *M.catarrhalis* (22.4%), and *S.aureus* (7.1%) were the most common pathogens. PICU stay and mechanical ventilation lasted longer in children with co-infections than children with negative cultures (9.1 vs 7.7 days, *p* = 0.04 and 8.1vs 6.5 days, *p* = 0.02).

**Conclusions:**

In this large study, bacterial co-infections occurred in more than a third of children requiring invasive ventilation for bronchiolitis and were associated with longer PICU stay and mechanical ventilation. These findings support a clinical trial of antibiotics to test whether antibiotics can reduce duration of PICU stay.

## Background

Bronchiolitis is a common respiratory condition in young children associated with a high morbidity. It is caused by viral infections, Respiratory Syncytial virus (RSV) being the commonest cause in hospitalized children [1]. The clinical picture of these infections ranges from mild symptoms to respiratory distress requiring hospitalization for supportive therapy [2]. About 10% of hospital admissions for RSV infection are so severe that invasive ventilation in a Pediatric Intensive Care Unit (PICU) is needed [3].

Although several risk factors for a more severe course of bronchiolitis have been identified, including cardiac disease, chronic lung disease, prematurity, this only partly explains why some children with bronchiolitis have severe respiratory failure and require mechanical ventilation [4].

Previous studies suggested that bacterial co-infections may be associated with a more severe course, though definitive evidence is lacking [5–8]. This is of particular interest as current treatment strategies for severe bronchiolitis are only supportive, whereas bacterial co-infections can be treated with antibiotics and thus could reduce morbidity.

The aim of this study is to investigate the prevalence and types of bacterial co- infection of the respiratory tract in children with bronchiolitis requiring invasive ventilation. We further investigated whether bacterial co-infection was associated with a prolonged duration of mechanical ventilation and PICU stay. Finally, we analysed whether bacterial co-infection can be predicted using clinical or laboratory markers such as C-Reactive Protein (CRP).

## Methods

### Design, setting and patients

This is a retrospective cohort study of children requiring invasive ventilation in the PICU of the Emma Children’s Hospital/Academic Medical Center Amsterdam, between December 2006 and November 2014. The PICU is a 14-bed, tertiary unit, serving the greater Amsterdam area in the Netherlands.

Patients less than 2 years of age who were clinically diagnosed with bronchiolitis and required invasive mechanical ventilation were included. In the Netherlands patients are referred to the PICU if they (are expected to) require (non) invasive ventilation. Diagnoses were scored on discharge by the attending PICU consultant and stored in an automated database containing all PICU admissions.

### Data collection

A standardised questionnaire was completed using data from the electronic patient chart that automatically recorded vital signs, ventilator settings; and collected medication given (Metavision®). An electronic patient chart was used to gather demographic data, previous medical history, physical examination on admission, laboratory results, chest X-ray on admission, microbiological results, and outcome parameters. The mechanical ventilation duration was defined as the cumulative period of invasive ventilation and non-invasive ventilation. If extubation failed the cumulative periods of (non-)invasive ventilation was used.

### Sampling and laboratory techniques

#### Viral

Nasal specimens were collected and used for DNA extraction and multiplex PCR as previously described [9]. The multiplex platform included influenza viruses (A and B), enterovirus, adenovirus, respiratory syncytial virus (A and B), rhinovirus (A-C), human metapneumovirus; parainfluenza viruses (1–4), parechovirus, human bocavirus and coronaviruses (hCoV; HKU1, NL63, 229E, and OC43). With every extraction and PCR, three controls were run. A minority of children had previously been tested in other hospitals for RSV using PCR or antigen tests. These data were used if multiplex results from our hospital were not available.

Viral testing was performed by the discretion of attending physicians. In some children no viral testing was performed at all.

#### Bacterial sampling

The cultures performed in this study were ordered by the attending physicians in case of clinical suspicion of a bacterial (super)infection. During the study period two sampling techniques were used: tracheal aspirates and mini-broncho-alveolar lavage [10]. Sampling was performed the endotracheal tube without previous installation of normal saline (tracheal aspirate) or by mini broncho-alveolar lavage. Tracheal aspirate was the primary sampling method. The mini-BAL was performed as part of an observative study on cytokine levels in bronchiolitis.

#### Bacterial cultures

Bacterial cultures were performed according to standard operating procedures at our laboratory. In brief, 10 μl of specimen were inoculated on standard media (CLED, Colombia, Sheep blood and Chocolate agars) and incubated for 1–2 days. Bacterial colonies were counted and identified by Vitek (Biomerieux) or Malditof (Bruker).

#### Other diagnostics

X-rays were scored and reported by attending radiologists. Plasma levels of C-reactive protein, were analyzed on Modular P800 and Modular Analytics E170 systems (Roche). Leukocytes were determined on an automated hematology cell counter.

### Definitions

To distinguish between colonisation and infection we used quantitative culture results, represented by the number of CFU per ml. We distinguished 3 categories based on the American Thoracic Society definitions in adults and Thorburn et al. [5, 11].

#### Bacterial counts

To distinguish between colonisation and infection we used the quantity of CFU/ml [5, 11] to distinguish 3 categories.

- *No bacterial growth:* no growth or growth of commensal bacteria only.
- *Low bacterial growth:* Presence of pathogens in the BAL <10^4^ CFU/ml or trachea aspirates <10^5^ CFU/ml.
- Bacterial c*o-infection:* Presence of pathogens in the BAL ≥10^4^ CFU/ml or trachea aspirates ≥10^5^ CFU/ml.

#### Timing

To distinguish between bacterial co- infections present on admission and those associated with ventilation we used the following definitions. *Early infections* (non-ventilator-associated) apply to cultures taken within 48 h of intubation, whilst *late infections* (ventilator -associated) refer to samples taken more than 48 h after intubation [11].

### Statistical analysis

Data was entered and analysed in an anonymised database SPSS 22.0 (SPSS Inc., Chicago, Illinois, USA). For comparison of continuous variables we used independent t – tests and for comparison of categorical variables Chi-square test and Fisher exact test were used. All p – value reported are two-sided and values less than 0.05 was considered significant. Potential predictors of duration of ventilation and PICU stay were assessed using univariate and linear regression analysis. Logistic regression was used to assess potential predictors of bacterial co-infection. Variables were included in multivariate analyses if *p*-values of univariate associations were < 0.1. Multivariate models included potential confounding factors (e.g. previous antibiotic use). The study adhered to the STROBE guidelines. Medical ethical approval was waived as it concerned retrospective analysis of anonymised patient data.

## Results

### Patient characteristics

Of 251 PICU admissions for bronchiolitis, 189 (75.3%) required invasive ventilation and were included in the study. The mean age was 2.9 months, 168 patients (88.9%) were less than 6 months of age and 108 (57.1%) were boys. Twenty-five patients had an underlying medical condition (13.3%) and 53 (31.0%) were prematurely born (Table 1). Data on antibiotic use prior to PICU referral was available in 101 children, 51 of these received antibiotics (50.5%). One patient died due to pulmonary hypertension.

**Table 1:** Patient characteristics of children with bronchiolitis requiring invasive ventilation

	*N* = 189
Demographic characteristics	
Boys	108 (57.1%)
Age (months)	2.91 ± 3.52
< 6 months of age	168/189 (88.9%)
Admission weight (kg)	4.91 ± 2.0
Born Premature^a^	53/171^b^ (31.0%)
Gestational age (weeks)	37.1 ± 4.05
Deaths^c^	1 (0.5%)
Underlying conditions	
Any underlying condition	25/188^b^ (13.3%)
Congenital Heart diseases	11/25^b^ (44.0%)
Down Syndrome	5 (20.0%)
Viral characteristics	
RSV	126/162^b^(77.8%)
2 or more viral infection	24/97^b^ (24.7%)
Clinical characteristics	
CRP max (mg/l)	85.0 ± 62.2
CRP 0–40 mg/l	55/188^b^ (29.3%)
40–100 mg/l	67/188^b^ (35.6%)
> 100 mg/l	66/188^b^ (35.1%)
Leukocytes highest value (× 10^9^ /l)	13.6 ± 6.4
Fever (> 38,5 °C)	4/149^b^ (2.7%)
Antimicrobial drug treatment	
Antibiotics started prior to PICU admission	51/101^b^ (50.5%)
Treatment started during PICU admission	159 (84.1%)
Cultures	
Cultured (BAL or TA)	167 (88.4%)

^a^: < 37 weeks

^b^data was only available for this proportion of patients

^c^Died from pulmonary hypertension

Data is either displayed as mean and standard deviation

RSV testing was performed in 162 (85.7%) patients and was detected in 126 (77.8% Table 1). In 97 (51.3%) children a full multiplex platform was performed and 24 children (24.7%) had presence of two of more viral pathogens (Table 1).

### Bacterial co-infection

In 167 of 189 (88.4%) patients one or more cultures (BAL or tracheal aspirates) were performed, whereas in 22 patients there was no culture performed (11.6%). Demographic data or underlying conditions were not different in these groups (data not shown).

Among the 167 cultured children, the overall occurrence of bacterial co-infections was 63 (37.7% of children cultured, or 33.3% of all 189 children). Low bacterial growth was found in 67 children (40.1%). In the 63 children with a bacterial co-infection 52 (82.5%) co-infections were detected within 48 h from intubation and were typed as early infection (Table 2). Thirty-four co-infections were detected by tracheal aspirate (54.0%), and 29 (46.0%) by mini-BAL. Patients who received antibiotics prior to PICU admission had more often negative cultures as compared to patients who did not receive antibiotics (43.2% versus 11.6%, *p* = 0.001).

**Table 2:** Prevalence of bacterial co-infection in children with bronchiolitis requiring invasive ventilation

	Cultured (*N* = 167)
Co-infection	63 (37.7%)
*Early co-infection*	*52 (82.5%)*
*Late co-infection*	*11 (17.5%)*
Low bacterial growth	67 (40.1%)
No growth	37 (22.2%)

Co-infection: Tracheal culture: > 10^5 CFU/ml or BAL: > 10^4 CFU/ml;

low bacterial growth: Tracheal culture: <10^5 CFU/ml or BAL < 10^4 CFU/ml;

negative: No growth or commensal flora

Early refers to samples collected < 48 h from intubation

Bacterial co-infections were found in 36.0% of 111 children with a proven RSV infection as compared to 21.9% in the RSV-negative group (7/32) and 66.7% in those who had no viral test performed (16/24; *p* = 0.002).

### Types of pathogens

In 63 children with a bacterial co-infection a total of 85 pathogens were isolated including *H. influenza (40.0%), S. pneumoniae (27.1%), M. catarrhalis (22.4%),* and *S. aureus (7.1%)* (Table 3). Enterobacteriaceae were only seen as late co-infection.

**Table 3:** Pathogens of co-infection in children with bronchiolitis requiring invasive ventilation

	Co-infection (*N* = 85)	Early co-infection (*N* = 69)	Late co-infection (*N* = 16)	*p*-value
*Haemophilus influenza*	34 (40.0%)	29 (42.0%)	5 (31.3%)	0,533
*Streptococcus pneumoniae*	23 (27.1%)	19 (27.5%)	4 (25.0%)	0,637
*Moraxella catarrhalis*	19 (22.4%)	17 (24.6%)	2 (12.5%)	0,286
*Staphylococcus aureus*	6 (7.1%)	4 (5.8%)	2 (12.5%)	0,280
*Enterobacteriaceae* ^a^	3 (3.5%)	–	3 (18.8%)	0,004

Co-infection: Tracheal culture: > 10^5 CFU/ml or BAL: > 10^4 CFU/ml; Early refers to samples collected < 48 h from intubation. ^a^Enterobacteriaceae: *E. coli* and *Enterobacter cloacae*

In 67 patients with low bacterial growth the distribution of pathogens was comparable to the co-infection group (data not displayed). Only *S. aureus* was more common in low bacterial growth as compared to the co-infection group (25.4% vs 7.1%, *p* = 0.02).

*S. pneumoniae* was found in 13.5% of RSV-positive patients (15/111), 3.1% in RSV-negative patients (1/32) and in 29.3% of patients who had no viral test performed group (7/24, *p* = 0.02).

### Duration of ventilation and PICU stay

Children with a bacterial co-infection required a mean duration of ventilation of 8.1 days compared to 6.5 days in children with a negative culture result (*p* = 0.02; Table 4). PICU length of stay was 9.1 and 7.7 days in children with and without a co-infection respectively (*p* = 0.04). No difference was noted in duration of ventilation nor PICU stay between the groups with co-infection and low bacterial growth. Early co-infections were not associated with a longer duration of ventilation or PICU stay as compared to children with negative cultures (7.5 vs 6.5, *p* = 0.12 and 8.5 vs 7.6, *p* = 0.21 respectively). Late co-infections were associated with a longer duration of ventilation and PICU stay (11.1 vs 6.5, *p* < 0.001 and 12.4 vs 7.6, *p* < 0.01).

**Table 4:** Duration of invasive ventilation and PICU stay in children with bronchiolitis

	Duration of ventilation (days)	*p*-value Uni−/multivariate	PICU stay	*p*-value Uni−/multivariate
			(days)	
Negative	6.5 ± 3,20		7.7 ± 3.55	
Co-infection	8.1 ± 3,47	0.021/0.016*	9.1 ± 3.45	0.044/0.054*
Low Bacterial Growth	7.3 ± 3,40	0.260/0.200*	8.7 ± 3.74	0.167/0.104*

*second *p*-values reflect regression analyses corrected for previous antibiotic use and age

### Predictors of a bacterial co-infection

A high CRP was associated with bacterial co-infection in a multivariate analysis including antibiotic use prior to PICU admission (*p* = 0.001, Table 5). A CRP level of 40 mg/L or more had a sensitivity of 85.7% and a specificity of 56.8% to detect a bacterial co-infection.

**Table 5:** Predictors of bacterial co-infection

	Negative (*N* = 37)	Co-infection (*N* = 63)	Low Bacterial Growth (*N* = 67)	*P* Value** univariate	*P* Value** Multivariate
Age (months)	4.1 ± 5.2	2.3 ± 2.6	3.0 ± 3.4	0.053	0.088
Fever (> 38,5 °C)	0 (0.0%)	3 (4.8%)	1 (1.5%)	0.394	
CRP (mg/l)*	57.6 ± 53.8	97.7 ± 51.8	102.6 ± 69.5	< 0.001	0.001
CRP 0–40 mg/l	21 (56.8%)	9 (14.3%)	12 (17.9%)	< 0.001	
40–100 mg/l	9 (24.3%)	28 (44.4%)	23 (34.3%)		
> 100 mg/l	7 (18.9%)	26 (41.3%)	32 (47.8%)		
Leukocytes* (10^e^9/ml)*	12.58 ± 4,72	14.67 ± 7.25	14.16 ± 6.79	0.085	–
Neutrophils* (10^e^9/ml)*	6.38 ± 4,45	8.93 ± 6.50	8.40 ± 6.41	0.048	–
Two viral infections	4/21 (19.0%)	6/23 (26.1%)	12/43 (27.9%)	0.578	
CXR*** Consolidation	15/36 (19.0%)	25/62 (40.3%)	33/66 (50.0%)	0.896	
Antibiotics before 1st culture	9/34 (75.0%)	21/59 (35.6%)	39/63 (61.9%)	< 0.001	0.000

* maximum value

** negative vs co-infection;- participated in the multivariate analysis, but removed

*** chest x-ray

## Discussion

In this large and concise study assessing the prevalence and relevance bacterial co-infections of the respiratory tract in children with bronchiolitis requiring invasive ventilation, bacterial co-infections were identified in at least a third of patients. In our population bacterial co-infections were associated with a longer duration of ventilation and PICU stay. CRP was associated with bacterial co-infection and may be used to identify children at increased risk.

### Presence of bacterial co-infection

In our study more than a third (37%) of the children with bronchiolitis requiring invasive ventilation had a bacterial co-infection. Potentially this number may have been higher as we used: a) a very strict definition of bacterial coinfection, ignoring 40% of children with low bacterial growth; b) 50% of children received antibiotics prior to sampling, which may have decreased detection of bacterial co-infections. We may also have overestimated the prevalence of bacterial co-infections as we used a clinical diagnosis of bronchiolitis whilst some children may have had a primary bacterial infection. This hypothesis is less likely though as the prevalence of bacterial co-infections in the RSV-positive group was comparable to the overall prevalence (36 and 37% respectively). The prevalence of bacterial co-infection is in line with previous studies that reported bacterial co-infections occurred in 20–45% of children with bronchiolitis admitted to PICU’s [1, 2, 5–8, 12–15]. Like our study, most data were retrospective and had similar limitations such as previous antibiotic use. A prospective study reported that 22% had a co-infection and 21% had low bacterial growth/possible co-infection [5]. Also, in this study more than 50% of children had received antibiotics prior to airway sampling.

Most co-infections were detected within 48 h, a cut off that is used to distinguish pre-existing infections from ventilator acquired pneumonias (VAP) [11]. Although it is not possible to distinguish pre-existing co-infections from infections acquired by our invasive techniques the fact that most co-infections occurred within these 48 h suggest that these infections are not likely introduced by intubation or colonization of endotracheal tubes.

### Bacterial pathogens

In our setting most bacterial isolates were common airway pathogens such as *H. influenza*, *S. pneumoniae*, *M. catarrhalis* and to a lesser extent *S. aureus*. This corroborates with data from bronchiolitis studies in Liverpool [5] and Zurich [6], however in our setting *S. pneumoniae* was more and *S. aureus* less common than in other studies. Enterobacteriaceae were solely identified as late co-infections; which is in line with previous data on VAP [16]. In our setting empirical treatment with amoxicillin (with or without clavulanic acid) would be appropriate for early infections, however in late co-infections an antibiotic with a broader coverage should be considered.

### Duration of ventilation and PICU stay

In this study we identified an association between bacterial co-infection and duration of PICU stay and mechanical ventilation. The difference was 2 days which can be considered clinically relevant. Three other studies reported on duration of ventilation and the presence of bacterial co-infections in children with bronchiolitis requiring ventilation. Kneyber et al. found a trend towards longer duration of mechanical ventilation in children with bacterial co-infections as compared to those without (14.3 vs. 10.6 days, *p* = 0.09) [7]; Thorburn et al. reported that bacterial infection contributed to longer mechanical support [5] and Hennus et al. confirmed that mechanical ventilation and bacterial co-infection were positively correlated [15].

The association between bacterial growth and a prolonged duration of mechanical ventilation suggests that bacterial co-infection could be of clinical importance. Alternatively, prolonged ventilation may predispose to increased prevalence of bacterial infections (ventilator associated pneumonia) [16]. In our study the difference in duration of ventilation was only significant in the late co-infections which corroborates with the latter theory. A similar trend was observed in the early co-infections which could suggest that both hypotheses are correct.

The association between co-infections and length of ventilation in our and other studies justify a trial to assess the role of antibiotics in reducing duration of ventilation and PICU stay in children with a bronchiolitis requiring invasive ventilation. Ideally the design of such a study should include a curative and preventive arm to evaluate both the role of pre-existing and ventilator associated bacterial pneumonia. Except for a study assessing the role of antibiotics to prevent PICU admissions [10], such a study has not been performed so far. The use of prophylactic antibiotics in ventilated patients has been studied in adults using selective digestive decontamination (SDD). D’Amico et al. showed that SDD given to adults admitted to ICU reduced VAP and overall mortality [17]. The data on the overall effect of all ventilated children admitted to PICU is conflicting however, but SDD appears to be effective in controlling respiratory infections [18]. No studies have been performed in the current subgroup of bronchiolitis patients admitted to PICU.

### CRP

In this study CRP was a useful predictor of bacterial co-infection, which corroborates with previous data [2]. That study used a much lower cut-off of 11 mg/L, which in practice does apply to most children admitted with a bronchiolitis and may not be very discriminative. Other studies did not find an association between CRP and bacterial co-infection in bronchiolitis patients [5, 10]. Unlike these studies we used the maximum CRP during admission, which may explain this discrepancy. CRP is known to have a twofold increase every 6 h, and therefor CRP dynamics could be used to guide antibiotic use in this population awaiting culture results.

### Limitations

Several limitations apply to our study, firstly we may have underestimated the prevalence of bacterial co-infections as we used a) a strict definition and b) the majority of the patients received antibiotics prior to sampling. These factors potentially may have contributed to an underestimation of the actual prevalence and underline that the number of bacterial co-infections in children with viral bronchiolitis in PICU is high. Alternatively we may have overestimated the role of bacterial co-infections in children with bronchiolitis as we recruited children based on the clinical discharge diagnosis of the attending PICU consultant. Although 4 of 5 children had a proven viral infection some children may have had a primary bacterial lower airway infection. This is less likely though as also in the group with proven RSV infection the co-infection rate was 36% as compared to 37% in the overall study.

Secondly, not every patient who was admitted to the PICU had a culture and cultures were performed based on clinically suspicion. This may mean that selection bias applied. However, the culture rate was nearly 90% and even if all children that were not cultured would have negative cultures still a third would have had a co-infection.

Thirdly, this was a retrospective and observational study, therefore we can only detect associations and not prove causal relationships. However, it justifies a prospective randomized controlled trial which could test the role of (curative or preventive) antibiotics to reduce duration of ventilation.

Fourthly, we have used different sampling techniques for bacterial cultures. However, we have applied strict definitions and corrected for this using the definition from Thorburn [5] et al. and international ATS guidelines [11].

### Clinical relevance

Until prophylactic or curative use of antibiotics have been tested in a double blind, randomized controlled trial, the use of antibiotics may be restricted to children with a suspicion of bacterial co-infection. Especially in young children and children with an increased CRP (> 40 mg/l) cultures should be taken and antibiotics covering normal airway pathogens should be considered. In case late co-infection is suspected empiric antibiotics should cover gram-negative bacteria and *S. aureus*. Future studies should prove if prophylactic or curative use of antibiotics could reduce duration of ventilation and PICU stay in bronchiolitis patients.

## Conclusions

In this large and concise study on bacterial co-infections in children with bronchiolitis we have found that bacterial infections are common, associated with prolonged ventilation and a raised CRP. Bacterial co-infections of the respiratory tract occurred in at least 37.7% of children with a bronchiolitis requiring invasive ventilation. In most cases bacterial co-infection was present within 48 h after intubation and was caused by common airway pathogens: *H. influenza, S. pneumoniae*, *M. catarrhalis.* Future studies should focus on the potential beneficial role of preventive or curative antibiotics to reduce duration of ventilation and PICU stay.

## Data Availability

The anonymized datasets used and/or analyzed during the current study are available from the corresponding author on reasonable request.

## Abbreviations

BAL: Broncho-alveolar lavages
CFU: Quantity of bacteria colony forming units
CRP: C-Reactive Protein
CXR: Chest X-Ray
PICU: Paediatric intensive care unit
SDD: Selective digestive decontamination
TA: Tracheal aspirates
VAP: Ventilator acquired pneumonia

## References

[1] Randolph AG, Reder L, Englund JA (2004). (sd). Risk of bacterial infection in previously healthy respiratory syncytial virus-infected young children admitted to the intensive care unit. Pediatr Infect Dis J.

[2] Fares M, Mourad S, Rajab M, Rifai N (2011). The use of C-reactive protein in predicting bacterial co-infection in children with bronchiolitis. North Am Med Sci.

[3] Bloomfield P, Dalton D, Karleka A (2004). Bacteraemia and antibiotic use in respiratory syncytial virus infections. Arch Dis Child.

[4] Wang EE, Law BJ, Stephens D (1995). Pediatric investigators collaborative network on infections in Canada (PICNIC) prospective study of risk factors and outcomes in patients hospitalized with respiratory syncytial viral lower respiratory tract infection. J Pediatr.

[5] Thorburn K, Harigopal S, Reddy V (2006). High incidence of pulmonary bacterial co-infection in children with severe respiratory syncytial virus (RSV) bronchiolitis. Thorax.

[6] Duttweiler L, Nadal D, Frey B (2004). Pulmonary and systemic bacteria co-infections in severe RSV bronchiolitis. Arch Dis Child.

[7] Kneyber M, Blusse van Oud-Albas H (2005). Concurrent bacterial infection and prolonged mechanical ventilation in children with respiratory syncytial virus lower respiratory tract disease. Intensive Care Med.

[8] Levin D, Tribuzio MD, Green-Wrzesinski T (2010). Empiric antibiotics are justified for children with respiratory syncytial virus lower respiratory tract infection presenting with respiratory failure: a prospective study and evidence review. Pediatr Crit Care Med.

[9] Jansen RR, Schinkel J, Koekkoek S (2011). Development and evaluation of a four-tube real time multiplex PCR assay covering fourteen respiratory viruses, and comparison to its corresponding single target counterparts. J Clin Virol.

[10] Kneyber MCJ, van Woensel JBM, Uijtendaal E (2008). Azithromycine does not improve disease course in hospitilized children with respiratory syncytial virus (RSV) lower respiratory tract disease: a randomized equivalence trial. Pediatr Pulmonol.

[11] American Thoracic Society; Infectious Diseases Society of America (2005). Guidelines for the Management of Adults with Hospital-acquired, Ventilator associated, and Healthcare-associated Pneumonia. Am J Respir Crit Care Med.

[12] Resch B, Gusenleitner W, Mueller WD (2007). Risk of concurrent bacterial infection in preterm children hospitalized due to respiratory syncytial virus infection. Acta Peadiatr.

[13] Hon KL, Leung TF, Cheng WY (2012). Respiratory syncytial virus morbidity, premorbid factors, seasonality, and implications for profhylaxis. J Crit Care.

[14] Ito H, Osamura T, Nakajima F (2007). survey of severe respiratory syncytial virus infection in Kyoto prefecture from 2003 to 2007. Pediatr Int.

[15] Hennus MP, van Vught AJ, Brabander M (2013). Mechanical ventilation drives inflammation in severe viral bronchiolitis. PloS One.

[16] Koenig SM, Truwit JD (2006). Ventilator-associated pneumonia: diagnosis, treatment, and prevention. Clin Microbiol Rev.

[17] D'Amico R, Pifferi S, Torri V, et al. Antibiotic prophylaxis to reduce respiratory tract infections and mortality in adults receiving intensive care. Cochrane Database Syst Rev. 2009;(Issue 4):CD000022. 10.1002/14651858.CD000022.pub3. 10.1002/14651858.CD000022.pub3 PMC7061255 19821262

[18] Ruza F, Alvarado F, Herruzo R (1998). Prevention of nosocomial infection in a pediatric intensive care unit (PICU) through the use of selective digestive decontamination. Eur J Epidemiol.

